# Evidence for IFNα-induced, SAMHD1-independent inhibitors of early HIV-1 infection

**DOI:** 10.1186/1742-4690-10-23

**Published:** 2013-02-25

**Authors:** Caroline Goujon, Torsten Schaller, Rui Pedro Galão, Sarah M Amie, Baek Kim, Kevin Olivieri, Stuart JD Neil, Michael H Malim

**Affiliations:** 1 Department of Infectious Diseases, King’s College London, 2nd Floor, Borough Wing, Guy’s Hospital, London Bridge, London SE1 9RT, UK; 2 Department of Microbiology and Immunology, University of Rochester Medical, Rochester, USA; 3 Infectious and Inflammatory Disease Center, Sanford-Burnham Medical Research Institute, La Jolla, USA

**Keywords:** HIV-1, Interferon, Restriction, Macrophages, SAMHD1, Vpx, Deoxyribonucleosides

## Abstract

**Background:**

Type I interferon (IFN) treatment of some cells, including dendritic cells, macrophages and monocytic THP-1 cells, restricts HIV-1 infection and prevents viral cDNA accumulation. Sterile alpha motif and HD domain protein 1 (SAMHD1), a dGTP-regulated deoxynucleotide triphosphohydrolase, reduces HIV-1 infectivity in myeloid cells, likely by limiting dNTPs available for reverse transcription, and has been described as IFNα-inducible. Myeloid cell infection by HIV-1 is enhanced by HIV-2/SIV_SM_ Vpx, which promotes SAMHD1 degradation, or by exogenous deoxyribonucleoside (dN) addition.

**Findings:**

SAMHD1 expression was not substantially influenced by IFNα treatment of monocyte-derived macrophages or THP-1 cells. The contributions of SAMHD1 to the inhibition of HIV-1 infectivity by IFNα were assessed through the provision of Vpx, exogenous dN addition, or via RNAi-mediated SAMHD1 knock-down. Both Vpx and dN efficiently restored infection in IFNα-treated macrophages, albeit not to the levels seen with these treatments in the absence of IFNα. Similarly using differentiated THP-1 cells, the addition of Vpx or dNs, or SAMHD1 knock-down, also stimulated infection, but failing to match the levels observed without IFNα. Neither Vpx addition nor SAMHD1 knock-down reversed the IFNα-induced blocks to HIV-1 infection seen in dividing U87-MG or THP-1 cells. Therefore, altered SAMHD1 expression or function cannot account for the IFNα-induced restriction to HIV-1 infection seen in many cells and cell lines.

**Conclusion:**

IFNα establishes an anti-HIV-1 phenotype in many cell types, and appears to accomplish this without potentiating SAMHD1 function. We conclude that additional IFNα-induced suppressors of the early stages of HIV-1 infection await identification.

## Findings

Type I interferon (IFN) treatment of some cell types, including macrophages, dendritic cells and the monocytic cell line THP-1, potently induces a block to HIV-1 infection at the level of viral DNA accumulation [1-4]. The identities and roles of participating IFN-induced anti-HIV-1 host factors are yet to be defined. The HIV-2/SIV_SM_ Vpx protein greatly increases the permissivity of myeloid cells to HIV-1 infection [5,6]. The protein sterile alpha motif (SAM) histidine/aspartic acid (HD) domain containing 1 (SAMHD1) was recently identified as a target for Vpx-induced proteasomal degradation in monocyte-derived macrophages (MDMs) and dendritic cells, as well as in quiescent CD4 T-cells [7-10]. SAMHD1 is a dGTP-regulated deoxynucleotide triphosphohydrolase that limits the pool of dNTPs available for reverse transcription, therefore reducing HIV-1 infection of myeloid cells [11,12]; for a short review see [13]. Interestingly, SAMHD1 has been reported to be IFNγ- and IFNα-inducible in human dendritic cells and monocytes, respectively [14,15]. And consistent with this, it was also shown that Vpx enhances HIV-1 infection of IFNα-treated monocyte-derived dendritic cells [16]. In this context, we sought to investigate further the role of SAMHD1 in IFNα-induced HIV-1 restriction.

**Figure 1:**
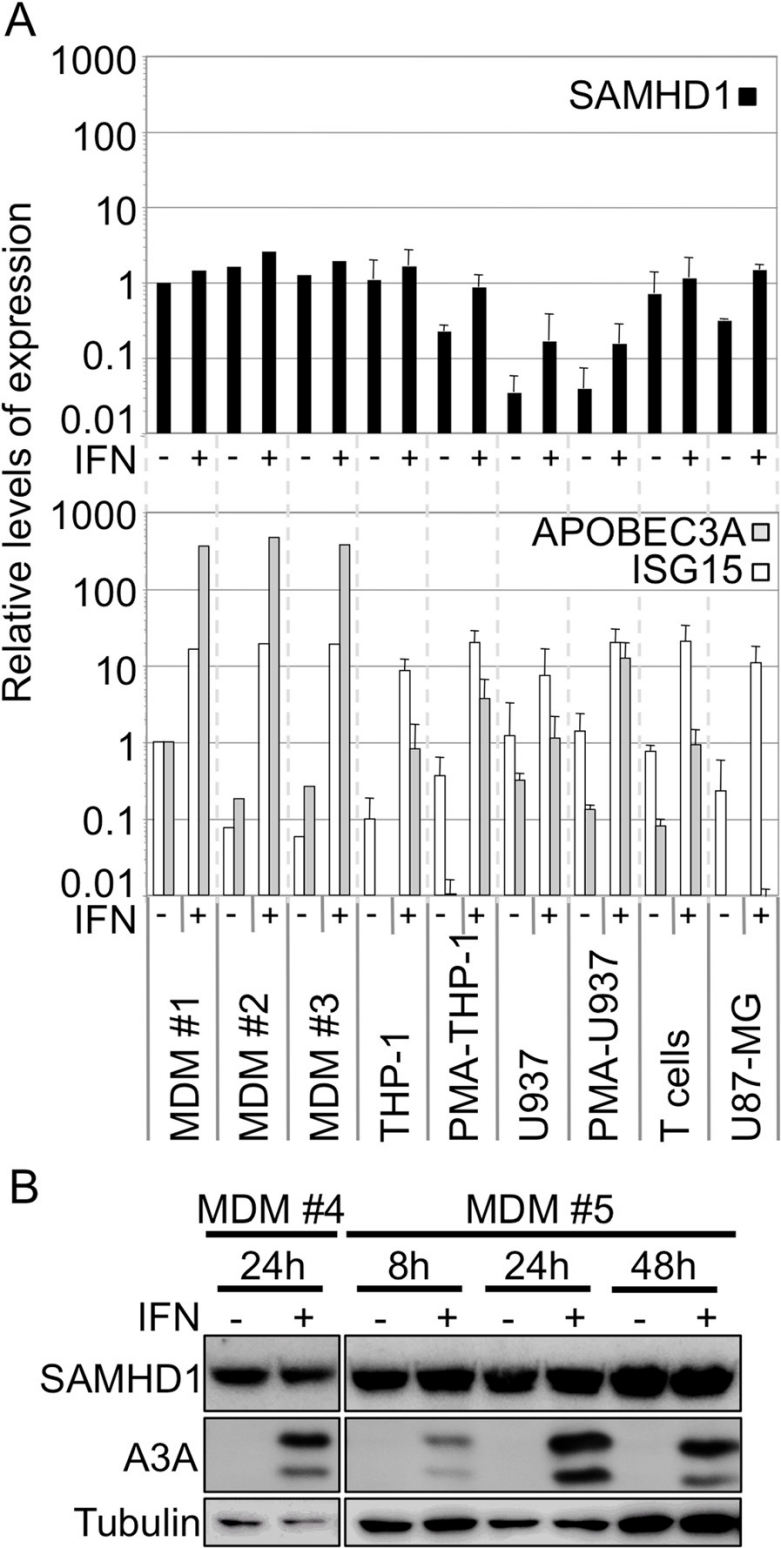
**SAMHD1 is induced poorly by IFNα in monocyte-derived macrophages. A**. 1 to 2 × 10^6^ monocyte-derived macrophages (MDMs) or primary CD4^+^ T cells (T cells), PMA-treated or dividing THP-1 and U937 cells, or U87-MG cells were incubated (or not) with 1000 U/ml IFNα for 24 h prior to total RNA extraction. cDNA was synthesized and the relative levels of SAMHD1 expression (upper panel), as well as two well-known ISGs, ISG15 and APOBEC3A (lower panel), were analyzed by RT-qPCR and the data were normalized to both GAPDH and β-actin expression. The graph shows the data obtained from 3 independent donors for MDMs (#1 #2 #3), and the mean values of relative expression in each cell type, obtained from 3 independent repetitions with cell lines or 3 different donors for CD4^+^ T cells. **B**. Western blot analysis. MDMs from 2 different donors were treated or not with 1000 U/ml IFNα for 24 h (donor #4), or for 8, 24 and 48 h (donor #5) before cell lysis. Protein levels of SAMHD1 and APOBEC3A (A3A) were determined, and tubulin served as a loading control.

To address the effects of type I IFN on SAMHD1 expression, we examined a variety of primary cell types and immortalized cell lines. MDMs and activated CD4^+^ T cells, both obtained from multiple donors, dividing and PMA-treated (differentiated) THP-1 and U937 cells, as well as U87-MG glioblastoma cells, were treated with 1000 U/ml IFNα for 24 h (for detailed description of experimental procedures, see Additional file 1). The cells were harvested for RNA extraction and RT-qPCR, or for western blot analysis (Figure 1). In MDMs, CD4^+^ T cells and dividing THP-1 cells, SAMHD1 was poorly IFNα-inducible (<2-fold increase in RNA abundance, Figure 1A; no difference was observed at the protein level for MDMs, Figure 1B), in contrast to two well-known IFNα-stimulated genes (ISGs), ISG15 and APOBEC3A [17-20]. In PMA-treated THP-1, untreated U87-MG, as well as in PMA-treated or dividing U937 cells, SAMHD1 mRNA levels were modestly upregulated by IFNα treatment (3- to 5-fold), possibly suggesting a cell type dependent effect of IFNα on SAMHD1 expression. Of note, U937 cells expressed more than one order of magnitude lower levels of SAMHD1 compared to MDMs or THP-1 cells, however IFNα treatment only increased SAMHD1 RNA levels by 3- to 5-fold. The IFNα-induced block to HIV-1 infection is observed in macrophages, PMA-treated and dividing THP-1 cells as well as in U87-MG cells, but not in U937 cells ([1]; and this manuscript), suggesting that induction of SAMHD1 expression by IFNα does not directly correlate with a block to HIV-1 infection. However, since SAMHD1 activity rather than abundance may be regulated by IFNα, we further investigated a possible role for SAMHD1 in the IFNα-induced suppression of HIV-1.

**Figure 2:**
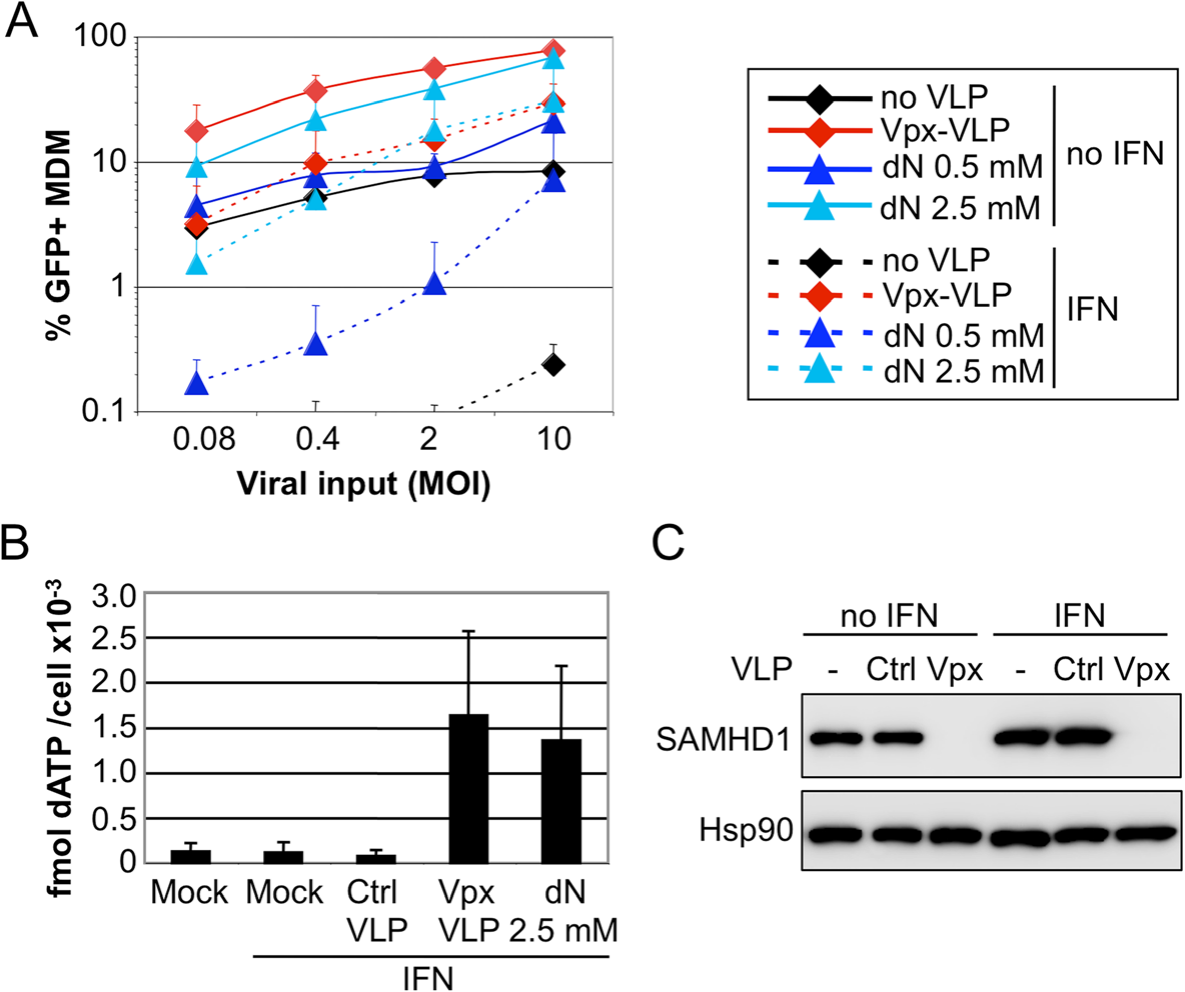
**Vpx-VLPs or exogenous dN treatment greatly enhance HIV-1 infection in IFNα-treated MDMs. A**. VSV-G pseudotyped HIV-1 derived GFP reporter virus was produced in 293T cells and used to infect control or IFNα-treated MDMs at different MOIs (0.08 to 10, as determined in 293T cells), in the presence or the absence of either Vpx-VLP or 0.5 mM or 2.5 mM deoxyribonucleosides (dN). Levels of infection were monitored using flow cytometry to measure the percentage of cells expressing GFP, and mean values from 4 independent experiments (using cells from 4 different donors) are shown. Notably, treatment of MDMs with the higher concentration of dN changed their morphology, with the cells becoming rounder and less adherent. We conclude that experiments using high dN doses must be analysed with caution. **B**. MDMs were treated or not with IFNα for 24 h and subsequently incubated or not with Ctrl-VLPs, Vpx-VLPs or dN (at 0.5 or 2.5 mM as indicated) for 16 h before lysis. Whole cell dATP was quantified using a single nucleotide quantification assay. Mean values from 4 independent experiments (using cells from 4 different donors) are shown. **C**. Western blot analysis. Cell lysates from MDMs (donor #4) were harvested 24 h post IFNα and Vpx-VLP treatment, and SAMHD1 expression was analysed by western blot; Hsp90 served as a loading control.

As both Vpx-containing virus-like particles (Vpx-VLPs) as well as exogenous deoxyribonucleosides (dN) relieve the SAMHD1-mediated HIV-1 block in myeloid cells [7,8,12], we evaluated the effects of these conditions on IFNα-treated MDMs (Figure 2). MDMs from 4 independent donors were treated or not with IFNα for 24 h prior to infection, and incubated with dN (0.5 or 2.5 mM), or with Vpx-VLPs, or left untreated (control). The cells were then infected with increasing amounts of a VSV-G pseudotyped HIV-1 encoding GFP reporter virus. The percentage of infected MDMs was enumerated by flow cytometry 2 days later (Figure 2A displays the mean data and Additional file 2: Figure S1A separates the data by donor). In agreement with previous reports [5,12,21], both Vpx-VLPs and exogenous dN increased HIV-1 infectivity in MDMs in the absence of IFNα (6- to 9-fold and 3- to 8-fold, respectively; Figure 2A). As we reported previously, IFNα treatment decreased HIV-1 infectivity in MDMs by at least 35-fold [1]. Strikingly, both Vpx and 2.5 mM dN efficiently stimulated HIV-1 infection in IFNα-treated MDMs (by more than 100-fold at the highest MOI). However, the levels of infection did not reach the levels observed without IFNα for either condition. Importantly, control VLPs that lacked Vpx did not have any positive impact on the efficiency of infection either in the absence or presence of IFNα (Additional file 2: Figure S1A).

The positive effect of both Vpx and dN correlated with significant increases in the intracellular dNTP pool (as shown by intracellular dATP measurement, Figure 2B displays the mean data and Additional file 2: Figure S1B shows the results for each donor). In agreement with this finding, Vpx efficiently induced the degradation of SAMHD1 (Figure 2C). Closer inspection of these data reveals that dN provision or Vpx addition promote infection to greater magnitudes in cultures treated with IFNα. While the molecular basis for this is not yet known, it is plausible that, although its expression is not increased, SAMHD1 activity might be potentiated in IFNα-treated MDMs. However, measurement of dATP levels in the presence or absence of IFNα did not reveal a significant difference in dATP concentrations, implying that the dNTP hydrolase activity of SAMHD1 is not measurably affected by IFNα treatment (Figure 2B and Additional file 2: Figure S1B). Alternatively, other restriction factor(s) might suppress HIV-1 infection more effectively when cellular dNTP concentrations are low. Of note, MDMs from different donors behave differently with respect to the rescue of HIV-1 infectivity following dN treatment, suggesting that donor-specific differences in SAMHD1 levels or activity contribute to differences in rescue by dN or Vpx (see donors 1 and 2, versus donors 3 and 4 in Additional file 2: Figure S1).

**Figure 3:**
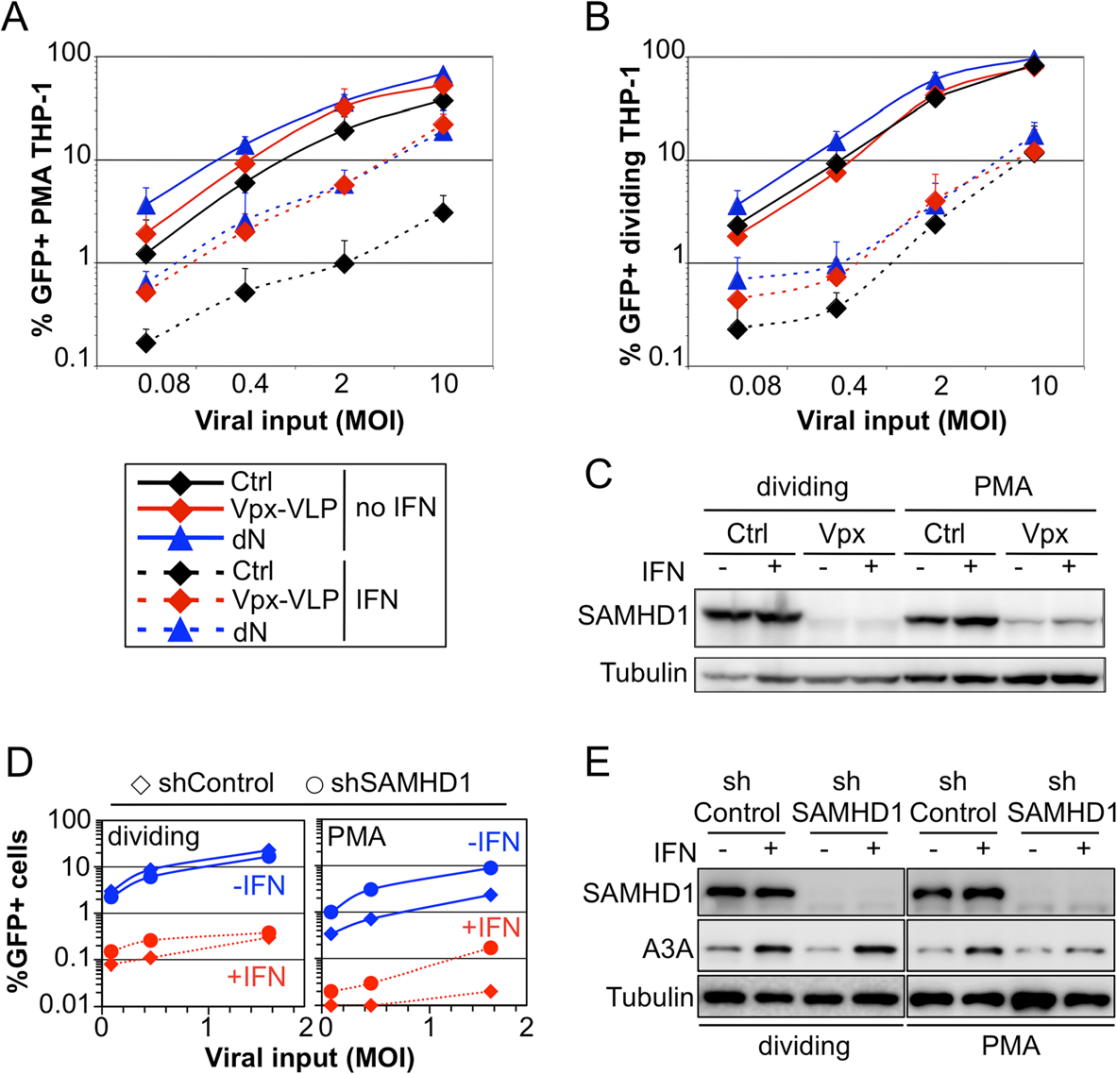
**Vpx-VLPs, exogenous dN treatment or SAMHD1 knock-down do not rescue the IFNα-induced block to HIV-1 infection in THP-1 cells. A**. and **B**. VSV-G pseudotyped HIV-1 derived GFP reporter virus was produced in 293T cells and used to infect control or IFNα-treated PMA-differentiated THP-1 cells (**A**), or dividing THP-1 cells (**B**) at different MOIs (0.08 to 10), in the presence or the absence of either Vpx-VLP or 0.5 mM deoxyribonucleosides (dN). Levels of infection were monitored using flow cytometry to measure the percentage of cells expressing GFP, and mean values from 3 independent experiments are shown. **C**. Western blot analysis. Cell lysates from THP-1 cells were harvested 24 h post IFNα and Vpx-VLP treatment, and SAMHD1 expression was analysed by western blot; tubulin served as a loading control. **D**. THP-1 cells expressing a control shRNA or shRNA targeting SAMHD1 were differentiated with PMA or not in the presence or absence of IFNα for 24 h. Cells were infected with 3 different doses of VSV-G pseudotyped GFP reporter virus for 48 h, and GFP positive cells were enumerated by flow cytometry. The data are representative of 3 independent experiments with two independent shRNAs against SAMHD1. **E**. Western blot analysis of parallel samples from D. Protein levels of SAMHD1 and APOBEC3A (A3A) were determined and tubulin served as a loading control.

We next evaluated the effects of dN and Vpx treatment on IFNα-treated THP-1 cells (Figure 3). PMA-differentiated THP-1 cells (Figure 3A) or dividing THP-1 cells (Figure 3B) were treated or not with IFNα for 24 h, and incubated with dN (0.5 mM), or with Vpx-VLPs, or left untreated (control) before infection with increasing amounts of a VSV-G pseudotyped HIV-1 encoding GFP reporter virus. In agreement with previous reports [12,21], both Vpx-VLPs and exogenous dN increased HIV-1 infectivity in PMA-THP-1 cells in the absence of IFNα (~2-fold; Figure 3A), but had no significant effect on HIV-1 infection of dividing THP-1 cells (Figure 3B). As reported previously, IFNα treatment decreased HIV-1 infectivity in THP-1 cells by at least 10-fold [1]. In PMA-treated THP-1 cells, both treatments partially increased HIV-1 infectivity following IFNα treatment, but had no significant effect in dividing THP-1 cells. Western blot analysis showed that Vpx-VLPs induced the degradation of SAMHD1 under all conditions (Figure 3C), indicating that IFNα-mediated suppression of HIV-1 infection in dividing and non-dividing THP-1 cells is maintained to significant degrees following SAMHD1 removal.

To investigate further the relationship between IFNα action and SAMHD1 function, we evaluated the effect of IFNα in THP-1 cells selectively depleted of SAMHD1 under dividing and differentiated culture conditions (Figure 3D). THP-1 cells were transduced with an HIV-1 shRNA vector targeting SAMHD1, or a control, and selected with puromycin. Dividing or PMA-treated shControl or shSAMHD1 THP-1 cells were treated with IFNα or not for 24 h, and then infected with different doses of VSV-G pseudotyped HIV-1 GFP vector (Figure 3D). As previously reported, reducing SAMHD1 protein levels increased the permissivity of PMA-treated (non-dividing) THP-1 cells by ~4-fold (Figure 3D) [7,15]. IFNα treatment decreased HIV-1 infectivity by more than one order of magnitude, irrespective of whether SAMHD1 was present or not, or whether the cells were dividing (Figure 3A and B). Although it is possible that low residual amounts of SAMHD1 were able to exert an effect, the data collectively suggest that SAMHD1 is, if at all, only a minor contributor to the IFNα-induced early block to HIV-1 infection in these cells. Of note, similar results were obtained in challenges with VSV-G pseudotyped full length HIV-1 (not shown).

**Figure 4:**
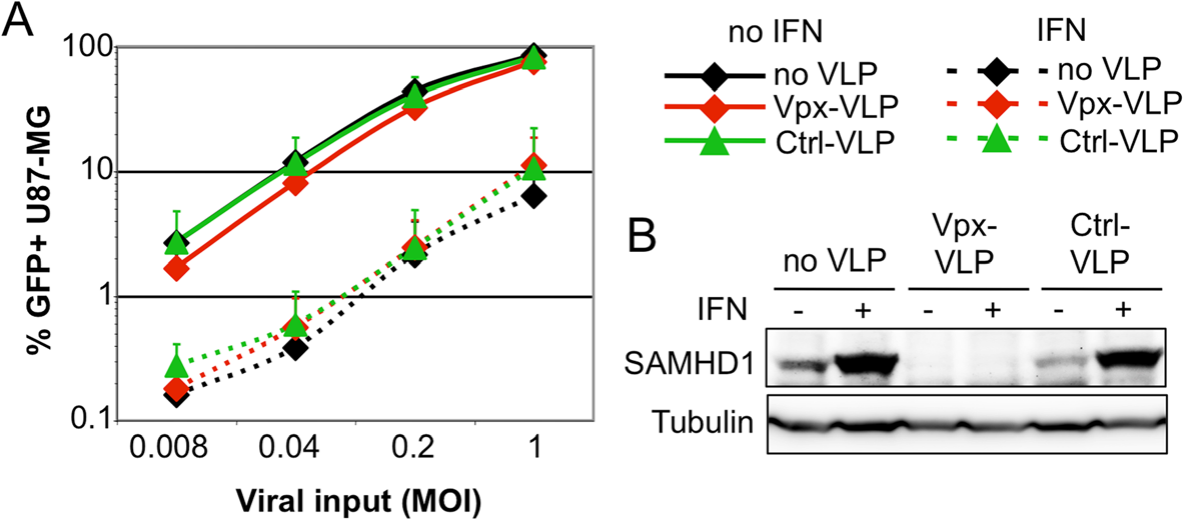
**SAMHD1 is induced by IFNα in U87-MG cells, but Vpx triggered degradation does not rescue IFNα-mediated HIV-1 suppression. A**. U87-MG cells were treated for 24 h with IFNα, or not, and incubated with no VLPs, Vpx-VLPs, or control VLPs (Ctrl-VLPs) and then infected with increasing amounts of GFP reporter virus (MOI 0.008 to 1), and GFP expression monitored as before. The mean values from 3 independent experiments are shown. **B**. Western blot analysis of a parallel samples from **A**. Protein levels of SAMHD1 were determined and tubulin served as a loading control.

SAMHD1 is highly expressed in MDMs and THP-1 cells, but to a lower extent in U87-MG cells and we found that IFNα treatment increased SAMHD1 expression in U87-MG cells to levels close to those observed in THP-1 or MDMs (Figure 1A) and western blot analysis confirmed increased protein levels (Figure 4B). To test for a cell-type specific activity of SAMHD1 after IFNα treatment we investigated whether Vpx-VLP treatment would rescue HIV-1 infectivity in IFNα-treated U87-MG cells. Cultures were treated or not for 24 h with IFNα, incubated either with Vpx-VLPs, with VLPs devoid of Vpx (Ctrl-VLPs) or left untreated, and then infected with increasing amounts of HIV-1 GFP vector (Figure 4A). As in myeloid cells (U937 cells excluded), IFNα treatment decreased HIV-1 infectivity in U87-MG cells by ~10-fold. Though SAMHD1 was efficiently degraded by Vpx-VLPs both in the absence or presence of IFNα (Figure 4B), the effects on HIV-1 infectivity were minor (maximum 2-fold increase, seen also with Ctrl-VLPs). In keeping with observations made in dividing THP-1 cells, this further demonstrates that SAMHD1 degradation does not necessarily correlate with a rescue of HIV-1 infection, and suggests that factors distinct from SAMHD1 are involved in the IFNα-induced block to HIV-1 infection in dividing U87-MG and THP-1 cells.

In this report, we used several primary and immortalized cell types to examine the relationship between SAMHD1 expression and function, and the inhibition of HIV-1 infection by IFNα. Taken together, our data reveal that SAMHD1, while previously established as an important regulator of HIV-1 infection [7-10], is not a major effector of IFNα-mediated HIV-1 restriction in a number of cell types and lines. In particular, by taking advantage of the ability of Vpx to degrade SAMHD1 [7,8], dN addition to bypass its deoxynucleoside triphosphohydrolase activity, or selective SAMHD1 knock-down, our findings indicate that IFNα is often able to suppress HIV-1 infectivity independently of SAMHD1 activity. In keeping with this, several of our experiments also showed that HIV-1 inhibitory phenotypes that bear the hallmarks of SAMHD1-mediated suppression are displayed in differentiated cultures of primary myeloid cells in the presence or absence of IFNα. Interestingly, Vpx and dN provoked a substantial increase in infection of IFNα-treated MDMs, which was greater than observed in untreated MDMs. This implies that either SAMHD1 function is at least partly responsible for the IFNα-mediated restriction in this cell type and/or its ability to reduce the pool of dNTPs is necessary for the action of other restriction factor(s). For example, we speculate that SAMHD1 may allow viral RNA or RNA/DNA reverse transcription intermediates to be specifically inhibited by IFNα-induced restriction factors, whilst the presence of Vpx or dN addition results in these structures being less abundant (or protected) owing to efficient reverse transcription. Lastly, it has been suggested that Vpx binds to APOBEC3A (A3A) possibly contributing to increased HIV-1 infectivity in monocytic cells [22,23]. Although we cannot rule out a role for A3A in the IFNα-induced block in MDMs described here, the rescue of HIV-1 infectivity by dN treatment argues against such a direct role. Moreover, A3A is not induced in U87-MG cells treated with IFNα, despite a potent block to HIV-1 infection. Our studies in U87-MG and THP-1 cells, where manipulation of SAMHD1 fails to overcome the effects of IFNα, allude to the existence of additional, possibly cell type specific, IFNα-inducible (and Vpx-independent) inhibitors of the early stages of HIV-1 infection. Validation of these models will require the identification of such cell-encoded factors.

## Abbreviations

Ctrl: Control;dATP: Deoxyadenosine triphosphate;dGTP: Deoxyguanosine triphosphate;dN: Deoxyribonucleoside;dNTP: Deoxyribonucleoside triphosphate;HIV-1: Human immunodeficiency virus type 1;IFNα: Interferon alpha;MDMs: Monocyte-derived macrophages;MOI: Multiplicity of infection;PMA: Phorbol-12-myristate-13-acetate;RNA: Ribonucleic acid;RNAi: RNA interference;SAMHD1: Sterile alpha motif (SAM) histidine/aspartic acid (HD) domain containing 1;shRNA: Short hairpin RNA;SIV: Simian immunodeficiency virus;VLP: Virus like particles;Vpx: Viral protein X

## Competing interests

The authors declare that they have no competing interests.

## Authors’ contributions

CG and TS designed the study, carried out the experiments and drafted the manuscript. RPG and SN helped design the study and provided reagents. SMA and BK did the dATP quantification. KO provided reagents. MHM designed the study and wrote the manuscript. All authors read and approved the final manuscript.

## Supplementary Material

Additional file 1 Material and methods. Click here for file

Additional file 2: Figure S1 Vpx-VLPs or exogenous dN treatment greatly improve HIV-1 infection in IFNα-treated MDMs. A. VSV-G pseudotyped HIV-1 derived GFP reporter virus was produced in 293T cells and used to infect control or IFNα-treated MDMs (from four different donors, donor #1 to #4) at different MOIs (0.08 to 10), in the presence or the absence of either Vpx-VLP or 0.5 mM or 2.5 mM deoxyribonucleosides (dN). Levels of infection were monitored using flow cytometry to measure the percentage of MDMs expressing GFP. B. Single nucleotide–incorporation analysis of dATP from MDMs treated or not with IFNα for 24 h and subsequently incubated or not with Ctrl-VLPs, Vpx-VLPs or dN (at 0.5 or 2.5 mM) for 16 h before lysis (+ Ctrl: + dATP; - Ctrl: no dATP). Click here for file
