# Supplementary material for: Evidence for IFNα-induced, SAMHD1-independent inhibitors of early HIV-1 infection
Source: Retrovirology. 2013 Feb 25;10:23. doi: 10.1186/1742-4690-10-23 (PMC3598776; doi:10.1186/1742-4690-10-23)
Supplement: Additional file 1 — Material and methods. [file 1742-4690-10-23-S1.docx]

**Material and methods**

**Cells and cell culture.**

Human primary CD4^+^ T cells and monocyte-derived macrophages (MDMs) were obtained from peripheral blood mononuclear cells (PBMCs) of healthy volunteer donors. Briefly, PBMCs were isolated using Lymphoprep (Axis-Shield) and monocytes were first obtained by positive selection using CD14 MicroBeads (Miltenyi Biotec). CD4^+^ T cells were then isolated from the remaining cells using the CD4^+^ T cells isolation kit II (Miltenyi Biotec). The purity of isolated cells was always >90% as judged by flow cytometric analysis of specific markers (CD4 and CD3 for CD4^+^ T cells or CD14 for monocytes). After 3 h in serum-free RPMI 1640 medium, monocytes were further differentiated into macrophages by culture for 4 to 6 days in RPMI 1640 supplemented with either 10% autologous serum or fetal bovine serum (FBS) (Gibco), and 100 ng/ml granulocyte-macrophage colony stimulating factor (GM-CSF) (R&D Systems). CD4^+^ T cells were stimulated for 20 h with 1 μg/ml phytohemagglutinin (PHA) (Oxoid) and 60 U/ml interleukin-2 (IL-2) (BD Pharmingen) prior to IFNα treatment. Human monocytic THP-1 and U937 cells were grown in RPMI 1640 medium, supplemented with 10% FBS. The differentiation of THP-1 and U937 cells was achieved by 24 h stimulation with 50 to 100 ng/ml phorbol-12-myristate-13-acetate (PMA) (SIGMA-Aldrich). Human glioblastoma U87-MG and 293T cells were maintained in complete DMEM plus 10% FBS. When indicated, IFNα (Universal type 1 IFN, PBL InterferonSource) was added at 1000 U/ml for 24 h prior to infection. When indicated, deoxyribonucleosides (dN, Sigma-Aldrich) were added at 0.5 or 2.5 mM at the time of viral challenge.

**GFP-expressing HIV-1 lentiviral vectors.**

The HIV-1-derived lentiviral vectors have been described previously [1, 2]. Vector stocks were obtained by polyethylenimine (PEI)-mediated triple transfection of 293T monolayers with constructs expressing Gag-Pol (p8.91), the miniviral genome bearing either a cytomegalovirus immediate-early promoter (CMV)-enhanced green fluorescent protein (eGFP) cassette (pRRL.sin.cPPT.CMV/eGFP.WPRE) or a spleen-focus forming virus (SFFV) promoter-eGFP cassette (pCSGW), and pMD.G [1]. Vpx containing- and control SIV_MAC251_ virus like particles (Vpx-VLPs and Ctrl-VLPs, respectively) were obtained by co-transfection of 293T cells with pMD.G and pSIV3^+^ [3] or pSIV3^+^vpx- (kindly provided by Dr Cimarelli) at a 1:2 DNA ratio. Culture medium was changed ~6 h after transfection, and virus- or VLP-containing supernatants were harvested ~36 h later.

GFP-encoding virus and VLP-containing supernatants were purified by ultracentrifugation through a sucrose cushion (20% w/v; 75 min; 4°C, 28,000 rpm using a Sorvall SureSpin630 rotor), resuspended in RPMI 1640 medium without serum and stored in aliquots at -80°C. Viral particles were normalized by HIV-1 p24^Gag^ ELISA, by RT ELISA (for SIV VLPs) and/or by determining their infectious titers on 293T cells. The multiplicity of infection (MOI) for vector stocks was determined by infecting a known number of 293T cells with standardized amounts of viral particles and evaluating by flow cytometry the percent of infected (GFP expressing) cells 2-3 days later. For instance, an MOI of 0.1 corresponds to the volume of virus necessary to obtain 10% GFP-expressing 293T cells.

**Viral infections.**

For viral infection, MDMs and THP-1 cells were plated at 1 to 2 × 10^5^ cells per well, in 96 or 48 well plates and U87-MG cells were plated at ~2 × 10^5^ cells per well in 24 well plates. Cells were usually treated with IFNα for 24 h prior to viral challenge. A volume of Vpx-VLPs or Ctrl-VLPs corresponding to an equivalent MOI of 1 (as determined by RT ELISA, in comparison to SIV GFP particles of known infectious titer) was added to the cells either 20 h prior to, or at the time of the infection. Infections were initiated by incubating with challenge viruses a few hours prior to replacing the medium. IFNα and dN were always re-added at the time of medium replacement. The efficiency of infection was analysed after 2 days by evaluating the percentage of GFP-expressing cells using flow cytometry (FACS Calibur, BD Biosciences).

**SAMHD1 silencing.**

SAMHD1 silencing was achieved using the CSRQ lentiviral vector bearing a puromycin resistance gene in addition to the shRNA coding sequence. pCSRQ was generated by substituting the GFP expression cassette in pCSGW with the shRNA cassette from pSIREN-RetroQ (Clontech), a vector driving shRNA expression through the human U6 promoter [4]. Suitable SAMHD1 silencing RNA target sequences were identified using the Clontech website http://bioinfo.clontech.com/rnaidesigner/sirnaSequenceDesignInit.do (shSAMHD1 5'-TGGAAATCTGTATGACATG; shCtrl 5'-TCGGCGCAGTCTAATTATA). Oligonucleotide sequences to generate short hairpin RNAs were designed using the Clontech website http://bioinfo.clontech.com/rnaidesigner/oligoDesigner.do, and ordered from Eurofins MWG (Ebersberg, Germany). Overlapping oligonucleotides encoding the shRNA were annealed and ligated between the *Eco*RI and *Bam*HI sites in CSRQ.

**Analysis of ISG induction.**

1 to 2 × 10^6^ cells were harvested 24 h after IFNα treatment or no treatment, and RNA was isolated using the miRNeasy kit with on-column DNase treatment (Qiagen). cDNA was synthesized with the High Capacity cDNA archive kit (Applied Biosystems) from normalized amounts (500 ng) of extracted RNA, and analyzed by Q-PCR using TaqMan® gene expression assays (Applied Biosystems) speciﬁc for human SAMHD1 (Hs00210019_m1), APOBEC3A (Hs00377444_m1), ISG15 (Hs00192713_ m1), β-actin (Hs99999903_m1) or glyceraldehyde-3-phosphate dehydrogenase (GAPDH; Hs99999905_m1). Triplicate reactions were run according to the manufacturer’s instructions using an ABI 7900HT sequence detection platform, and the RQ software (Applied Biosystems) was used for subsequent analysis. For relative quantiﬁcation, samples were normalized to both GAPDH and β-actin mRNA content.

**Immunoblot analysis.**

Cell samples were lysed in sample buffer (200 mM Tris-HCl pH 6.8, 5.2 % SDS, 20 % glycerol, 0.1 % bromphenol blue, 5% β-mercaptoethanol), resolved by sodium dodecyl sulfate-polyacrylamide gel electrophoresis, and analyzed by immunoblotting using primary antibodies specific for SAMHD1 (mouse monoclonal [1F9], Abcam), tubulin (DM1A, Sigma-Aldrich), HSP90 (Santa-Cruz Biotechnology, Inc.) or APOBEC3A [5], followed by secondary horseradish peroxidase-conjugated anti-mouse or anti-rabbit immunoglobulin antibodies and chemiluminescence (ECL+ western blotting substrate, Pierce).

**Whole-cell dATP quantification.**

Monocytes were plated at 1.5 × 10^6^ cells per well in 6 well plates and differentiated into MDMs for 4 days as described above. MDMs were then treated or not with IFNα for 24 h prior to Ctrl-VLP, Vpx-VLP, or dN treatment for 16 h. MDMs were then washed in 1X PBS and lysed in 65% methanol. Whole-cell dATP contents were measured using a single nucleotide incorporation assay, as described [6].

**References**

1. Naldini L, Blomer U, Gallay P, Ory D, Mulligan R, Gage FH, Verma IM, Trono D: **In vivo gene delivery and stable transduction of nondividing cells by a lentiviral vector.** *Science* 1996, **272:**263-267.

2. Bainbridge JW, Stephens C, Parsley K, Demaison C, Halfyard A, Thrasher AJ, Ali RR: **In vivo gene transfer to the mouse eye using an HIV-based lentiviral vector; efficient long-term transduction of corneal endothelium and retinal pigment epithelium.** *Gene Ther* 2001, **8:**1665-1668.

3. Mangeot PE, Duperrier K, Negre D, Boson B, Rigal D, Cosset FL, Darlix JL: **High levels of transduction of human dendritic cells with optimized SIV vectors.** *Mol Ther* 2002, **5:**283-290.

4. Schaller T, Ocwieja KE, Rasaiyaah J, Price AJ, Brady TL, Roth SL, Hue S, Fletcher AJ, Lee K, KewalRamani VN, et al: **HIV-1 capsid-cyclophilin interactions determine nuclear import pathway, integration targeting and replication efficiency.** *PLoS Pathog* 2011, **7:**e1002439.

5. Koning FA, Newman EN, Kim EY, Kunstman KJ, Wolinsky SM, Malim MH: **Defining APOBEC3 expression patterns in human tissues and hematopoietic cell subsets.** *J Virol* 2009, **83:**9474-9485.

6. Diamond TL, Roshal M, Jamburuthugoda VK, Reynolds HM, Merriam AR, Lee KY, Balakrishnan M, Bambara RA, Planelles V, Dewhurst S, Kim B: **Macrophage tropism of HIV-1 depends on efficient cellular dNTP utilization by reverse transcriptase.** *J Biol Chem* 2004, **279:**51545-51553.
