# Supplementary figures and images for: Evidence for IFNα-induced, SAMHD1-independent inhibitors of early HIV-1 infection
Source: Retrovirology. 2013 Feb 25;10:23. doi: 10.1186/1742-4690-10-23 (PMC3598776; doi:10.1186/1742-4690-10-23)

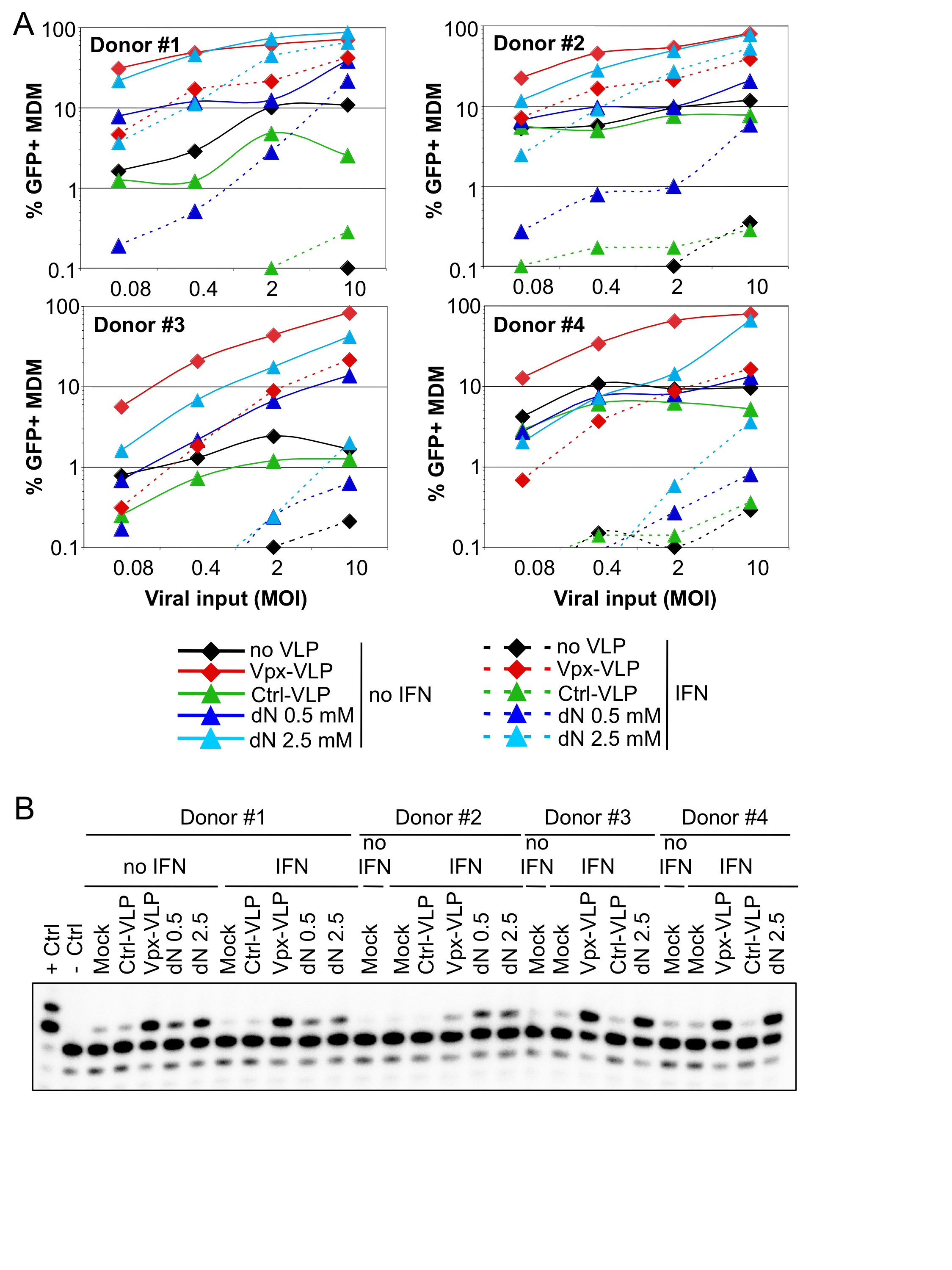

Supplement: Additional file 2: Figure S1 — Vpx-VLPs or exogenous dN treatment greatly improve HIV-1 infection in IFNα-treated MDMs. A. VSV-G pseudotyped HIV-1 derived GFP reporter virus was produced in 293T cells and used to infect control or IFNα-treated MDMs (from four different donors, donor #1 to #4) at different MOIs (0.08 to 10), in the presence or the absence of either Vpx-VLP or 0.5 mM or 2.5 mM deoxyribonucleosides (dN). Levels of infection were monitored using flow cytometry to measure the percentage of MDMs expressing GFP. B. Single nucleotide–incorporation analysis of dATP from MDMs treated or not with IFNα for 24 h and subsequently incubated or not with Ctrl-VLPs, Vpx-VLPs or dN (at 0.5 or 2.5 mM) for 16 h before lysis (+ Ctrl: + dATP; - Ctrl: no dATP). [file 1742-4690-10-23-S2.png]
